# Reducing residential mortgage default: Should policy act before or after home purchases?

**DOI:** 10.1371/journal.pone.0200476

**Published:** 2018-07-19

**Authors:** Yifei Wu, Jeffrey H. Dorfman

**Affiliations:** 1 International CCAE Modeling/Econometric Indicator/Risk Management, Citi, Wilmington, Delaware, United States of America; 2 Department of Agricultural and Applied Economics, The University of Georgia, Athens, Georgia, United States of America; Universitat Jaume I, SPAIN

## Abstract

We examine two possible approaches to reducing residential mortgage default using a dynamic model of heterogeneous infinitely-lived agents acting optimally subject to uninsurable idiosyncratic earnings shocks and systemic house price shocks. We find higher down payments are very effective in minimizing residential mortgage foreclosures, even in periods of house price declines and recessions. In contrast, the length of the credit exclusionary period for people who experience bankruptcy or foreclosure has a much smaller impact on mortgage defaults. Thus, it is much more effective to prevent mortgage default before the mortgage closes than to pressure homeowners not to default once they are in financial trouble. This also suggests a major aspect of credit scores and credit policy is non-productive and punitive, harming people in return for little societal gain.

## Introduction

The 2007–2009 recession was at least partially caused by a major contraction in the housing sector and a significant increase in mortgage delinquency and default rates. Thanks to concentrated investments in real estate, the U.S. banking system suffered greatly from the substantial number of mortgage foreclosures and household bankruptcies that arose due to the housing downturn that began in 2005. As the real estate market remained depressed and the financial sector struggled to work through the credit crunch magnified by the bursting of the housing bubble, 2010 alone saw more than 1 million houses enter foreclosure and 1.5 million households file for bankruptcy [1]. The tremendous economic and social devastation wrought by the mortgage crisis highlights the importance of understanding the impact that policy levers have on household behavior as it relates to foreclosures and bankruptcy during a house price bust period. Specifically, can we implement policies that reduce residential mortgage defaults in the future, thus lowering the risk of another financial crisis?

In this paper we look at the efficacy of two potential policy levers: the size of down payment required and the length of time someone who defaults on his mortgage is subsequently excluded from the credit markets. These two policies are of particular interest for two reasons. First, both can be and have been varied and are within control of the government policy makers for at least the most part. Second, one policy effects home buyers before the home purchase while the other becomes relevant after the purchase and, then, only if the borrower begins to experience financial distress.

This paper applies a new heterogeneous agent dynamic model of rational utility-maximizing households to study the impact of two common credit policy levers (down payments and the credit exclusionary period) on both mortgage defaults and personal bankruptcy filings. We solve a dynamic model of a household that can purchase a house with a mortgage or continue renting and must simultaneously decide how much to consume and borrow from credit cards in each period. With uninsurable idiosyncratic earnings shocks and systemic house price shocks, homeowners can find themselves in unforeseen financial difficulties. At such a point they have two channels for default: file for bankruptcy or go into mortgage foreclosure (short sales are also an option in our model, as described below). Understanding the linkage and interaction between these two default behaviors and our two policy levers is crucial for explaining the observed aggregate empirical data and reforming our credit markets to minimize the recurrence of such debt crises. Thus, our paper makes a contribution to this literature by including both secured and unsecured debt [2] and making home price shocks systemic to better mimic the recent bursting of the real estate bubble [1].

These decisions on the model do matter, and the literature contains many possible choices. Some previous studies of mortgage default are based on option theory [3, 4], in which the default option will be exercised if it is deeply in the money. This traditional “strategic default” theory assumes that borrowers default on their mortgage to maximize (minimize) their financial gains (losses), even though they still have enough liquidity to pay the mortgage. In these models, negative home equity is a necessary but not sufficient condition for default, and if equity drops below a threshold level the homeowner will go into foreclosure.

Another strand of literature believes that foreclosure behavior is triggered not only by negative home equity, but also by other factors—a “double trigger.” For example, [5] and [6] argue that both negative home equity and a household liquidity constraint “double trigger” mortgage foreclosures. In opposition, [7] points out that over 80% of mortgage defaulters were above water in the 1998 and 2001 Surveys of Consumer Finance, so default behavior is not caused solely by income shocks and negative home equity. It has also been debated whether a change in policy towards more recourse loans led to a lower aggregate mortgage foreclosure rate [2, 8, 9, 10].

A direct investigation the effect of low down payments on the rise in foreclosures in the late 1990s can be found in [2]. However, their model abstracts from unsecured debt and bankruptcy, focusing primarily on mortgage loans. A joint analysis of foreclosure and bankruptcy with a one-period mortgage and unsecured debt and given steady state house prices is performed in [1]; agents face idiosyncratic house price shocks, but there are no aggregate house price shocks.

In addition to the studies with structural models, existing empirical studies provide discussion of more factors which might change homeowners’ propensity to default on mortgages. Personal bankruptcy filings under both chapter 7 and chapter 13 served to decrease the five-year home foreclosure rate by 1.7% and 19.1% [11]. Foreclosure has a spillover effect on nearby house prices which can create a feedback loop, causing more defaults [12]. From a macro point of view, [13] summarized many financial and regulatory factors that led to the last US mortgage crisis.

All these previous works inform the more general model used here. We begin with the framework developed by [14] to study strategic credit card default and modify it by introducing housing, mortgage and bankruptcy elements to study mortgage default. Our resulting model accurately simulates actual household behavior from 1985 to 2014, closely matching the historical data across a variety of economic and credit market statistics. We then use this structural model to simulate borrower behavior under different economic conditions, with different levels of down payments, and different length credit exclusionary periods. These simulation results allow us to demonstrate which levers can effectively reduce credit defaults during periods of financial stress.

In terms of contribution within this literature, our paper is one of the few to include both mortgage and unsecured debt in the same model. We also believe we have the most flexible modeling of housing price shocks, which allows us to accurately simulate the rise and fall in house prices that created the recent mortgage market meltdown. In combination, this makes our model more general and more useful for policy analysis than other credit models in the literature to date. Our model’s generality allows us to examine whether it is easier to prevent mortgage defaults in advance (with a higher down payment) versus discouraging defaults once the borrower begins to seriously consider such an action (with the threat of a period of exclusion from the credit markets).

The remainder of the paper is structured as follows. In section II, the theoretical structure of our model is presented along with a brief description of the computational methods used to solve the model. Section III presents the parameter values used to calibrate the model and provides empirical results to demonstrate that the model tracks recent historical data well. The model results, both in steady state simulations and under conditions similar to the recent real estate market collapse are discussed in section IV. Finally, section V concludes the paper.

## The model economy

The main elements of the model are that a) the economy is comprised of infinitely-lived agents facing both exogenous employment and house price shocks in each period, b) all agents act each period in order to maximize the expected present value of lifetime utility, although there is a stochastic hurdle to be cleared before an agent changes her status, c) agents have access to mortgage loans and credit card debt as long as they are deemed credit worthy, and d) agents can either default on their mortgage (go through foreclosure) or file for bankruptcy in each period. While a growing literature in macroeconomics investigates the problem of mortgage foreclosure in a general equilibrium setting in which interest rates are determined endogenously [15], we are interested in variables under more direct control of credit market policy makers; therefore, we fix interest rates at their average value for this period. Following are the details.

### Representative agents

At the end of each period, all households possess net savings *s*, with *s* < 0 indicating debt and *s* > 0 representing liquid savings. Every household initially has access to unsecured debt and can borrow up to a certain credit limit *b*. If the household is in an employed state, *i* = 1, it receives normalized income $\bar{y}=1$; if in an unemployed state, *i* = 0, it receives an unemployment benefit $\underline{y}<1$.

Households that do not own a house are called renters (*k* = 0). Renters can choose each period to buy a house, declare bankruptcy, or continue renting. Agents are exposed to a house market price shock (*H*) each period, so by waiting there is a chance of buying a home later at a different, potentially lower, price (*h*). To simplify, agents can only purchase at most one house each and must finance the housing purchase with a down payment and a 30 year fixed rate mortgage. While not all home buyers use 30 year mortgages, it is the most common mortgage and the one most used by the riskiest borrowers and those with the least home equity. Thus, simplifying our model to just 30 year mortgages likely still captures most defaulters accurately. Also, neither early mortgage payoffs nor refinancing are allowed. Therefore, for 30 years after the housing purchase, the household will have an installment payment obligation, during which it is referred to as homeowner (1 ≤ *k* ≤ 30). In each period, a homeowner can choose to declare bankruptcy, default on the mortgage, sell the house, or continue paying the mortgage. All unsecured debt is discharged in bankruptcy, but the household will immediately be flagged as an unworthy one and barred from borrowing for some years (τ) as a penalty (the exclusionary period). Similarly, after going through a non-recourse mortgage foreclosure, the agent will also be credit unworthy and barred from buying a house for τ years.

After 30 years, those who own a house and are finished with mortgage payments are called homeowners with no mortgage (*k* > 30). Beginning in the following year, such agents have a certain probability of dying, causing the house to be sold and resulting in a change in the household head. Though households have infinite life in this model, their houses are not assumed to be inherited by the next generation. This assumption, essentially blocking inherited wealth, simplifies the model and avoids the disappearance of debt over time.

#### The strategic decisions under uncertainty

Each agent is in one of the states above at the start of each period. To make the optimal strategic decision, each agent considers her value function for every decision available to her and chooses the decision that has the maximal value function. For example, a credit worthy homeowner can choose between continuing to make mortgage payments, selling the house, filing for bankruptcy, or defaulting on the mortgage.

Each household in this model maximizes a state-contingent value function of a current state variable over an infinite time horizon. The agent’s dynamic decision problem in a particular state is characterized by a Bellman Equation which is subject to a budget constraint. The value functions for all seven possible states are shown in the S1 Appendix to this paper. The set of decisions an agent faces are shown below for two different states to make the assumed decision process concrete. The remaining cases can easily be constructed from the different value functions given in the S1 Appendix.

Households make stochastic decisions in this model based on the relative values of the current expected lifetime utility of each choice. In particular, after each agent has solved the value function for each possible decision, the probability of changing states is given by an exponential function of the difference between the value function of a new state and the value function of the current state. That is, if an agent’s expected lifetime utility in her current state is denoted by V and the corresponding value in a potential new state is represented by W, then she will decide to move from the current to new state (such as going from renter to homeowner) with probability of change equal to 1−e−(W−V)μ. If no decision’s value function is above that for the current state, the probability of change is 0; if two or more decisions have expected utility gains, the probability of change described above applies only to the one with the largest expected gain. Note the function above works well in this context because it is non-decreasing and right-continuous in its domain.

Introducing uncertainty in the decision making process provides a better approximation to how real households make decisions since few of us actually solve full dynamic programming models before deciding whether or not to buy a house or enter foreclosure. Instead, we are implicitly assessing the likelihood of each option being our best choice. Also, most people have a bias toward remaining in their current state, which this approach mimics. The value of the parameter μ controls how big this bias is, with larger values of μ requiring a larger expected utility gain to reach a specific probability of changing states.

Because the value functions several states’ Bellman Equations do not have closed form solutions, they were solved numerically using dynamic programming and the *fminbnd* function in the MATLAB platform [16]. The riskless asset domain from–b to 4 is divided into 200 equally spaced grid points and a linear interpolation is used to represent the value function [8]. The procedure to find a solution is as follows:

Step 1: Make an initial guess as to the solution of the value function *V*_0_(s).
Step 2: Iteratively update V using a single-variable function minimization algorithm based on the golden section search and parabolic interpolation. The value at each grid point is independently updated each iteration and linear interpolation of the updated grid is used to approximate *V*_*t*+1_,
  $$V_{t+1}(s)=\max_{\mathrm{s'}}F[V_{t}(s)].$$ (1)
Step 3: When *V* reaches convergence, *V*_*T*+1_(s) ≈ *V*_*T*_(s), then the iteration is halted and the problem is solved.

#### The strategic decision of the worthy renter

The worthy renter has three options. First, he can continue to be a worthy renter, the value function of which is

$$V^{R}(s;i;j=0)=\max_{c}\{u(c)+\beta \sum_{i^{\prime }}p_{{i,i}^{\prime }}V^{R}(s^{\prime };i^{\prime };j\prime =0)\}$$ (2)

subject to $\frac{s^{\prime }}{1+r}+c+\xi \hat{H}=s+y(i)$

$$s^{\prime }\geq -b$$

$$r=\left\{ \begin{array}{cc} r_{b} & s\prime <0\\ r_{s} & s\prime \geq 0 \end{array}\right..$$

**Fig 1:**
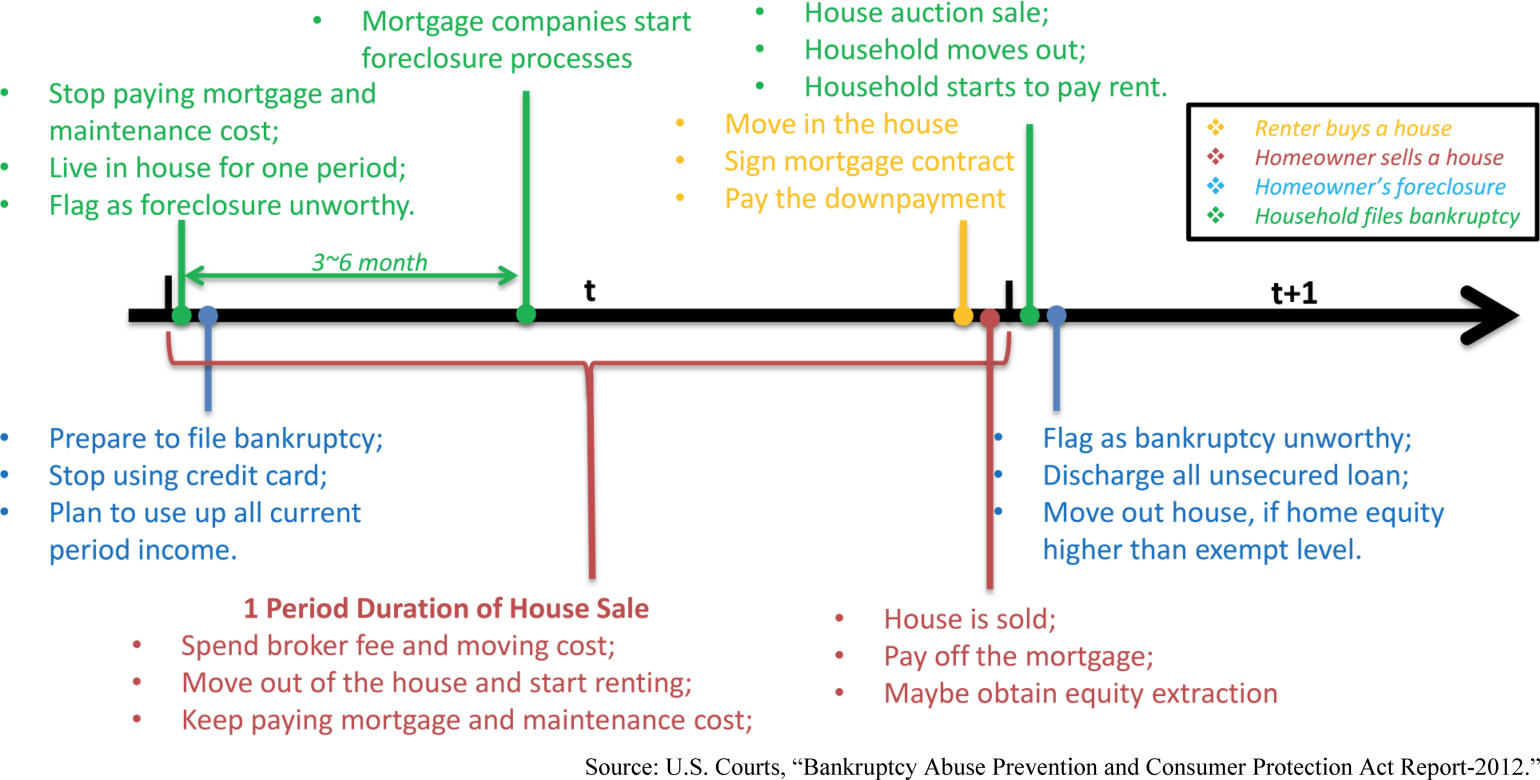
The timeline of events within a period.

Second, the renter can buy a house by obtaining a mortgage. Although this decision is made at the beginning of each period, due to the time required to find, buy, and obtain a mortgage on a house, the house is assumed to be purchased at the end of the period (Fig 1). Therefore, in that period he is liable for both a down payment and rent. At the beginning of the next period, the renter is a first-year homeowner.

Finally, when a worthy renter is trapped deeply in debt, he can also declare bankruptcy. In this model, it is assumed that households can only file Chapter 7 bankruptcies, primarily because bankruptcy filing under chapter 7 far exceeds any other type. Specifically, in 2012 the number of Chapter 7 bankruptcy filings accounted for 69.12% of personal bankruptcy filings. Under Chapter 7, households have no reason to save money or repay the debt during the bankruptcy filing period because they expect all unsecured debt to be discharged at the beginning of the next period. Thus, it is assumed that they will spend as much as they can and begin with a zero balance in the next period. To avoid being accused of fraud, renters cannot accumulate more than σ in debt that period. The value of σ is assumed to be 15 percent of income. The value function if bankruptcy is chosen is given by

$$W^{R,bankrupcy}(s,i,j=0)=u(c)+\Theta +\beta \sum_{i^{\prime }}p_{{i,i}^{\prime }}V^{R,B}(s^{\prime }=0,i^{\prime },j=1)$$ (3)

subject to

$$c+\xi \hat{H}=\max\left(s,0\right)+y(i)+\min\left(\sigma ,b+s\right).$$

Here, bankruptcy behavior incurs a pure utility loss, represented as social stigma Θ.

#### Strategic decision of the worthy homeowner with mortgage

The worthy homeowner has four options: (1) keep paying her mortgage, (2) declare bankruptcy, (3) default on the mortgage, or (4) sell the home. First, if a homeowner keeps paying her mortgage, her value function $V_{k}^{h}$ is given by equation (A5) in the S1 Appendix.

Second, if the homeowner chooses to declare bankruptcy while paying the mortgage, the homeowner will consume as much as she can knowing the unsecured debt will be discharged next period. The bankruptcy trustees’ interest in selling the house depends on the homestead exemption (Ξ) and the amount of home equity at the time of the bankruptcy filing. If home equity is greater than the homestead exemption, the bankruptcy trustee will sell the house, pay off the mortgage, and reimburse the household for the exemption; otherwise, the homeowner can keep her house and mortgage. All houses are assumed to be auctioned at the beginning of the next period, ending the bankruptcy process. The house market price in the next period is uncertain; hence, the current market house price is used to estimate the probability of the next period price. This implies a value function of

$$W_{k}^{h,bankrupcy}(s,i,j=0)=u\left(c\right)+\Theta +\beta \sum_{{Η}^{\prime }}q_{H,H^{\prime }}{\tilde{V}}_{k+1}\left(H\right)\;\forall \;0<k\leq 30$$ (4)

subject to $c+\kappa \hat{H}+\Psi (h,D,r_{m})=\max\left(s,0\right)+y(i)+\min\left(\sigma ,b+s\right).$

The contingent value function of the house market price is given by:

$${\tilde{V}}_{k+1}\left(H'\right)=\{\begin{array}{lll} \sum_{i^{\prime }}p_{i,i^{\prime }}V_{k+1}^{h,B}\left(s^{\prime }=0,i^{\prime },j^{\prime }=1\right) & \mathrm{if} & \left(1-\chi \right)H^{\prime }-\Omega \leq \Xi \\ \sum_{i^{\prime }}p_{i,i^{\prime }}V^{R,B}\left(s^{\prime }=\Xi ,i^{\prime },j'=1\right) & \mathrm{if} & \Xi <\left(1-\chi \right)H^{\prime }-\Omega \leq \Xi -s\left(1+r\right)\\ \sum_{i^{\prime }}p_{i,i^{\prime }}V^{R,B}\left(s^{\prime },i^{\prime },j'=1\right) & \mathrm{if} & s^{\prime }=\left(1-\chi \right)H^{\prime }-\Omega +s\left(1+r\right)\leq \Xi \end{array}$$ (5)

Here, Ω = Ω(*h*,*D*,*r*_*m*_,*k* + 1) represents the outstanding mortgage debt in year k+1 of the mortgage. In some very rare cases (the third ${\tilde{V}}_{k+1}$ equation), the bankruptcy trustee sells the house, pays off the mortgage debt in full, reimburses the household for the homestead exemption, and pays off the unsecured debt, again reimbursing the household if any cash is left over.

Third, the homeowner can allow foreclosure on her home by stopping payments on the mortgage and maintenance at the beginning of the period [9]. In 3 to 6 months, the homeowner will be flagged as a foreclosure unworthy renter but she can still live in the home until the house auction sale at the end of this period [17]. The impact of foreclosure duration on default behavior is tested in [17]. While the expected length of the foreclosure process may play a role in homeowners’ decisions, this is not a factor that would be easy for policy makers to regulate, so we simply assume all foreclosures in our model take one period (year). The lengthy foreclosure process saves one year of rent for this household Fig 1. The lifetime utility of the foreclosure homeowner is given by:

$$W_{k}^{h,foreclosure}(s,i,j=1)=\max_{c}\{u(c)+\beta \sum_{i^{\prime }}p_{{i,i}^{\prime }}V^{R,F}(s^{\prime },i^{\prime },j\prime =2)\}$$ (6)

subject to

$$\frac{s^{\prime }}{1+r_{s}}+c=s+y(i)+max[0,(1-\phi )H-\Omega (h,D,k+1)]$$

$$\frac{s^{\prime }}{1+r_{s}}\geq \min\left(0,s\right).$$

Finally, instead of just walking away from the foreclosed home, the homeowner can also choose to short sell her house. If the homeowner chooses to short sell the house, she faces double housing costs for that period because she will need to move out of the house, prepare it for sale, and rent another house at the beginning of the period. At the end of the period, the house will be sold, and the mortgage debt will be paid off. When the homeowner decides to terminate this mortgage contract, the current period’s cost and benefit will be compared directly as follows. The homeowner will choose foreclosure when the condition

$$max[0,(1-\chi )H-\Omega (h,D,r_{m},k+1)]-\kappa \hat{H}-\Psi (h,D,r_{m})<max[0,(1-\phi )H-\Omega (h,D,r_{m},k)\times (1+r_{m})]+\xi \hat{H}$$ (7)

holds; otherwise, she will short sell the house and extract the home equity.

## Calibration

### Model economy

A number of parameters must be specified to complete the model; these parameters are used to make the model match the actual behavior of the U.S. economy over the recent past.

In this study, two economic states are considered: a normal and a recession economy. Because the expected duration of a recession economy is about 2 years (while recessions as defined by negative GDP growth rarely last two years, unemployment is slower to recover and that is the more relevant variable for mortgage default; thus, we use a two year “recession” period), all households in a recession economy have a prior probability of 0.33 for the economy to return to a normal state in the next period, making the median recession last two (year-long) periods in our model. Thus, the value functions of the recession economy are dependent on the corresponding value functions of the normal economy,

$$V_{t}^{recession}=u(c)+(0.33V_{t+1}^{normal}+0.67V_{t+1}^{recession}).$$ (8)

Three recent recession periods have been recorded by the National Bureau of Economic Research: July 1990 to March 1991, March 2001 to November 2001, and December 2007 to June 2009. U.S. unemployment rate and duration data were obtained from BLS. Based on these data, we set the unemployment rate (*γ*) in the normal and recession economies to 5% and 9%, respectively.

Besides the unemployment rate, there are two other parameters used to differentiate the two states of the economy: the unemployment carryover rate *p*_00_ and the annual expected income of unemployed agents. As a part of the (2x2) transition probability matrix of the employment Markov chain, *p*_00_ represents the probability of an unemployed agent remaining unemployed in the next period. Given the unemployment carryover rate, the unemployment rate, and a probability distribution *f(x)* summarizing the probability of the length of a spell of unemployment, the transition probability matrix can be determined by

{p00=∫52+∞f(x)dxp01=1−p00p11=γp01(1−γ)p10=1−p11. (9)

**Fig 2:**
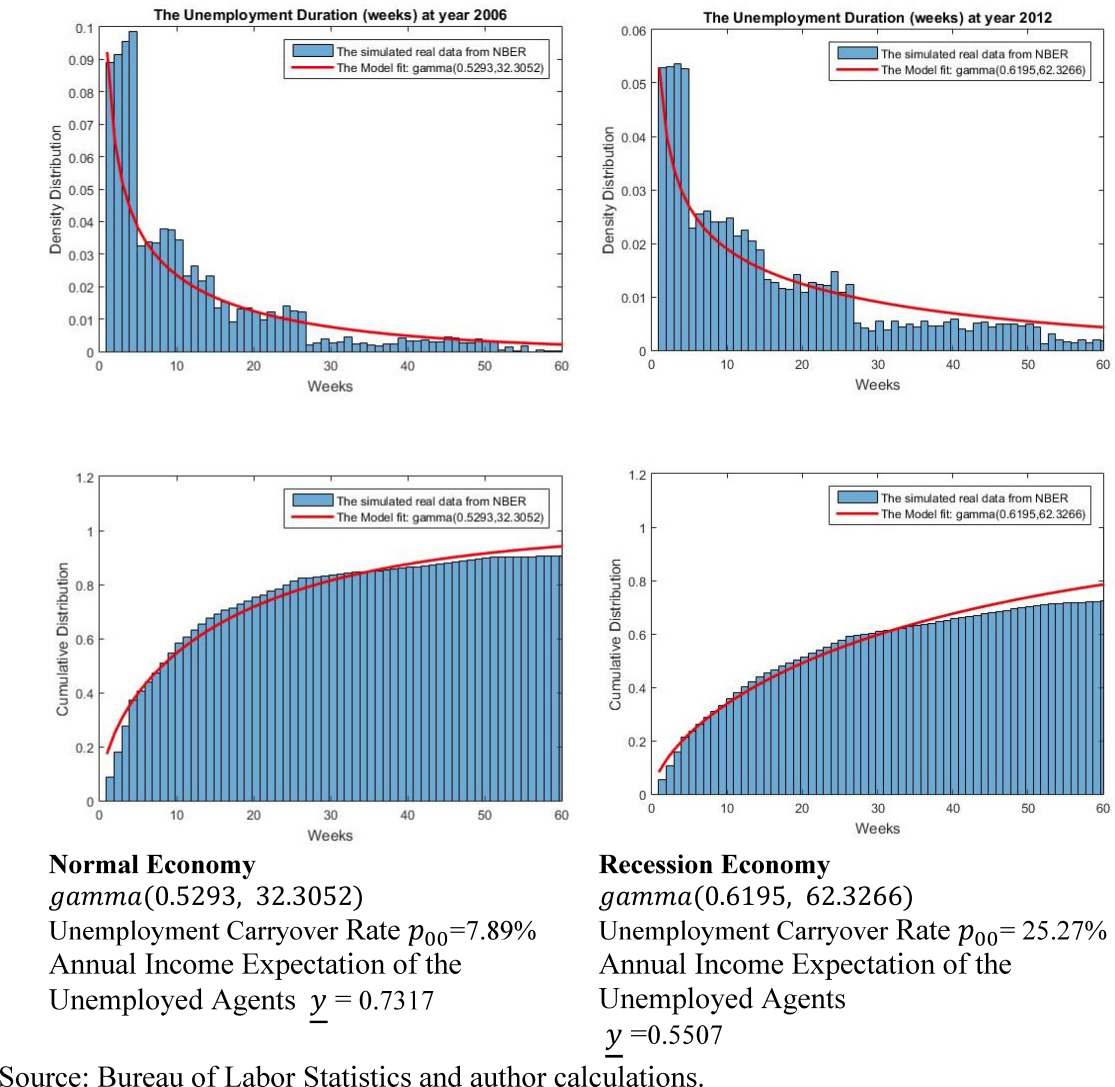
The distribution of unemployment duration.

The annual income of employed agents in both states of the economy is normalized to 1; meanwhile, the unemployed agents expect their annual income to be $\underline{y}$. In the United States, unemployment benefits generally pay eligible workers between 40–50% of their previous pay. That is the major reason why [14] assumed the unemployed annual income expectation to be 0.4. However, the standard time-length of unemployment compensation is 6 months; once this 6-month time period elapses, payments cease. In order to precisely estimate the annual income expectation of unemployed agents as well as the unemployment carryover rate, the distribution of unemployment duration is approximated from the BLS data. Excluding the data from the ambiguous small recession period, the data from 1994 to 1999 and 2005 to 2007 are used to approximate the distribution in the normal economy. Two histograms are drawn to depict both the probability density and cumulative distributions of unemployment duration in both the normal and the recession economy (Fig 2). To match the histogram shape, a gamma distribution is fit to the data and its two parameters are estimated by maximum likelihood. Then, the annual expected income of unemployed agents in both economic states can be computed by the following equation:

$$\underline{y}=\int_{0}^{26}\left[0.4\frac{x}{52}+1\left(1-\frac{x}{52}\right)\right]f_{X}\left(x\right)dx+\int_{26}^{52}\left[0.4\frac{26}{52}+1\left(1-\frac{x}{52}\right)\right]f_{X}\left(x\right)dx.$$ (10)

The results of the parameter estimation are displayed in Fig 2. In the normal economy, the unemployment carryover probability is 7.87%, more than three times lower than the 25.27% in the recession economy. The annual expected income of an unemployed agent is reduced from 0.7317 in the normal economy to 0.5507 in the recession economy. These are the values used in our model.

#### Aggregate house prices

We simplify away the choice of housing size, assuming only one house size is available. All houses have an underlying value normalized to three times the annual income of an employed household which approximates the ratio of household income and house prices in the 2013 Survey of Consumer Finance for median income households ($46,700 and $125,000, respectively).

As mentioned earlier, the price fluctuation of house prices around their underlying value is the second major source of shocks in our model. This study models house price shocks using a nine state, discrete time Markov chain. The transition matrix Q of this Markov chain is calibrated using the real U.S. Case-Shiller Home Price Index without seasonal adjustment from 1890 to 2013. Housing is very different from most financial assets and commodities which are universally priced and comparable across regions, in that housing is fixed in place which regionalizes the market and makes values harder to discover. Before selling their houses, homeowners can only imperfectly predict the market value of their houses using information from sources such as the Case-Shiller National House Price Index or from comparable neighborhood sales. According to [18], this uncertainty about current house market prices has proven to be important in alleviating the aggregate foreclosure rate in the mortgage crisis. In our model, all households are assumed to predict their current market house price solely from the change in the aggregate house price index in the last three periods. Additionally, each household’s prediction is stochastically selected according to current house price conditional probabilities.

To obtain these probabilities without loss of generality, house prices were simulated over 100 million periods. Homeowners’ expectations about their current house price levels are estimated by the sample proportions of past house price changes conditioned on the pattern of price changes in the previous three years. Similarly, [19] assumed that agents in the economy have heterogeneous beliefs about fundamentals that drives house price.

Next, the log of the Case-Shiller Home Price Index is decomposed into trend and cyclical components using the nonparametric Hodrick–Prescott filter. Specifically, assume the log home price series variable *z*_*t*_ is composed of a trend component, *x*_*t*_, and a cyclical component, *w*_*t*_; that is, *z*_*t*_ = *x*_*t*_ + *w*_*t*_. Given a positive value of *λ*, there is a trend solution that minimizes:

$$\min_{x}\left\{\sum_{t=1}^{T}{\left(z_{t}-x_{t}\right)}^{2}+\lambda \sum_{t=2}^{T-1}{\left[\left(x_{t+1}-x_{t})-(x_{t}-x_{t-1}\right)\right]}^{2}\right\}.$$ (11)

**Fig 3:**
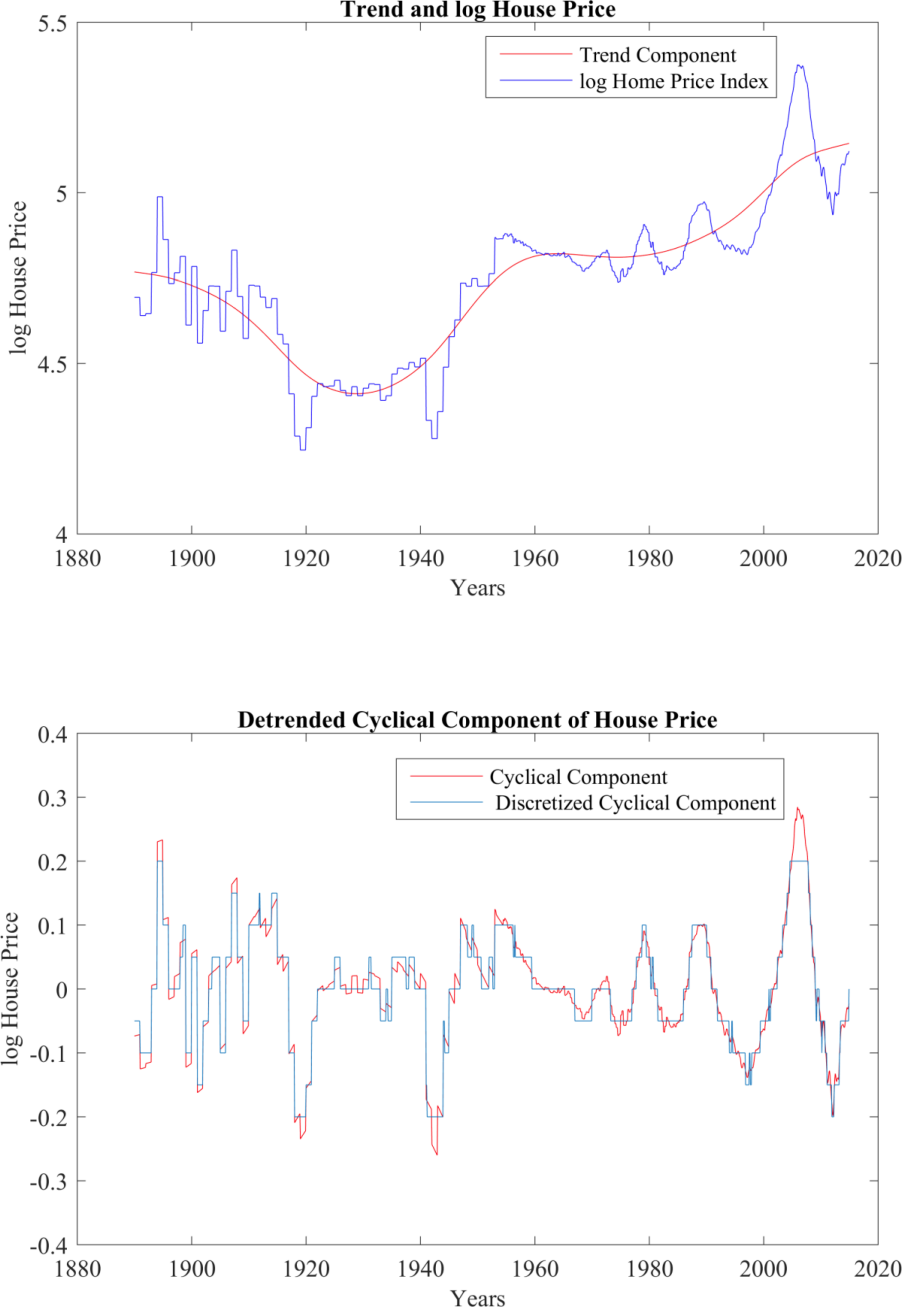
The decomposition of real US case-shiller home price index, without seasonally adjusted. Source: S&P Dow Jones and author calculations.

The multiplier *λ* represents the sensitivity of the trend component to short term fluctuations. The higher the *λ* value, the smoother the trend component is and the longer term are the fluctuations captured by the cyclical component. This study requires that the stochastic process captures a longer period price cycle. For this reason, *λ* is set to 3 × 10^7^ by trial and error, which is relatively higher than [20], who used 129,600 for monthly data. Fig 3 shows the result.

The stochastic house price process is discrete, with the cyclical stochastic changes being set at -20%, -15%, -10%, -5%, 0%, 5%, 10%, 15% and 20% of the current house value. The transition probabilities are estimated by the sample proportion:

$${\hat{q}}_{ij}=\frac{n_{ij}}{\sum_{k=1}^{9}n_{ik}}$$ (12)

where the denominator, $\sum_{k=1}^{9}n_{ik}$, is the total number of data observations in state *i*, and the numerator, *n*_*ij*_, is the number of times that state *i* values move to state *j* in the next period.

#### Rent and other costs related to housing

Rent does not always follow the house price cycle, a situation particularly true during and after the recent recession. Rather, rents ($\xi \hat{H}$) tend to be proportional to the underlying value of housing ($\hat{Η}$), not the market house price (*H*). The price-to-rent ratio was estimated to be 12 from two Zillow Research datasets: the median of the value of all homes per square foot and the median rent of all homes per square foot. The rent cost proportion parameter *ξ*, the reciprocal of the price-to-rent ratio, is, thus, 0.0833. Annual upkeep ($\kappa \hat{H}$) is assumed proportional to the underlying value of housing. We set the proportion parameter *κ* to be 0.035, which includes maintenance (2%), property taxes (1%), furniture replacement, pest control, etc.

Houses in foreclosure are sold at an average 28% discount [21]; meanwhile, other forced sales (e.g., short sales, tax sales) only have a 3% to 7% discount. In this study, the sale value discounts are set to *ϕ* = 0.28 and *χ* = 0.06 in foreclosure and short sale cases, respectively.

#### Preferences

The utility function of a household with respect to consumable durable goods is taken to be in the constant elasticity of substitution (CES) family. In housing and rental studies, a commonly used utility function is the constant relative risk aversion function nested with Cobb-Douglas preferences over consumption and housing services [1]:

$$u(x,H)=\frac{{\left(c^{\pi }h^{1-\pi }\right)}^{1-\alpha }-1}{1-\alpha }$$ (13)

Here, *α* is the constant relative risk aversion coefficient and H is house price. In this function *π* is calibrated to match the share of annual housing expense in total consumption. It is worth noting that the *h* in this function does not denote the housing price, but annual housing expenses.

Previous research has employed a general homeowner’s utility function [22]:

$$u(c,Η)=\frac{c^{1-\alpha }-1}{1-\alpha }+\gamma \frac{{\hat{H}}^{1-\alpha }-1}{1-\alpha },\;\gamma >0$$ (14)

where *γ* is relative desirability of housing. However, in this study, the utility derived from housing is fixed because both income and house size are normalized for either renter or homeowner. To adjust for this and for our more general model, we employ an isoelastic flow utility function based on the framework from [22] and modify it to account for homeowners and renters:

$$u(c)=\frac{c^{1-\alpha }-1}{1-\alpha }+\delta \frac{H^{1-\alpha }-1}{1-\alpha }I(own)$$ (15)

where *I*(*own*) is an indicator variable which equals one if the agent owns a home in the current period and zero otherwise. This utility flow only accounts for the emotional utility gain of home ownership depending on the current house market price [23] described δ as a homeowner’s emotional attachment to the house; this parameter is internally calibrated in our model. The constant relative risk aversion α is set to 3, which is standard in this field [24, 14]. The bequest motive η is set by 0 for simplicity.

#### Financial intermedia

The interest rate of unsecured debt (*r*_*d*_), mortgage debt (*r*_*m*_), and saving (*r*_*s*_) are set based on recent empirical averages (*r*_*d*_ = 12%, *r*_*m*_ = 6% and *r*_*s*_ = 3%), which places them somewhat above current rates for mortgages and savings but still reasonable. The 2007 Survey of Consumer Finance [25] reported that the median total credit limit per family was about $18,000, which was 36% of median family income and used as our unsecured credit limit. We set the credit exclusionary period as *τ* = 7 in the base case, corresponding to the current average 7 years without access to the credit market as punishment for a credit default. Strictly speaking, filing for bankruptcy should not affect one’s credit score, but in practical terms, the credit reporting agencies are allowed to report bankruptcy history for up to 10 years. For simplicity, this study assumes that both bankruptcy and mortgage default will result in a credit exclusionary period of *τ* years. The bankruptcy homestead exemption varies greatly in different states, and it is set equal to one year’s median income in the base case, Ξ = 1. In the base case, the down payment ratio *D* is set at 10%, which is very standard in the literature [5].

#### Remaining parameter calibration

The parameters whose values have been set so far are either fairly standard in the literature or can be estimated directly or indirectly from the data (Table 1). The remaining parameters will be calibrated so the model matches a set of empirical macroeconomic evidence (Table 2). These remaining parameters are the discount factor *β*, social stigma Θ, death rate of household heads *ω*, emotional attachment to the house *δ*, and the exponential mean parameter *μ*. Theoretically speaking, all five parameters jointly determine simulation outputs due to the complexity of this heterogeneous agent model. However, to reduce the optimization dimension, the discount factor *β*, social stigma Θ, and death rate of the household head *ω* are independently calibrated first.

**Table 1:** Externally calibrated base case parameters.

Parameter	Value	Description	Source
*α*	3	Coefficient of risk aversion	(Lopes, 2008)
*γ*_*normal*_	5%	Unemployment rate in normal economy	(Wang and Miranda, 2015)
*γ*_*recession*_	9%	Unemployment rate in recession economy	(Wang and Miranda, 2015)
*η*	0	Bequest motive	(Low, 2015)
*κ*	0.035	Maintenance cost proportion	
*λ*	3 × 10^7^	Hodrick–Prescott filter multiplier	
*ξ*	0. 0833	Rent cost proportion	Zillow Research Data
*ρ*	0.33	Economy reinstatement rate	
*σ*	0.15	Debt increase limit during bankruptcy filling	Base case assumption
*τ*	7	Credit exclusionary period	Base case assumption
*ϕ*	0.28	Foreclosure value discount	(Campbell et al., 2011)
*χ*	0.04	None-foreclosure value discount	(Campbell et al., 2011)
Ξ	1	homestead exemption	Base case assumption
*b*	0.36	Credit limit	(Wang and Miranda, 2015)
*r*_*s*_	3%	Risk-free asset rate of return.	3 month treasury bond
*r*_*m*_	6%	Mortgage interest rate	Market Quote
*r*_*b*_	12%	Interest rate on unsecured debt	Market Quote
*D*	15%	the downpayment ratio	Base case assumption

**Table 2:** Internally calibrated parameters.

Description	Parameter	Value	Target	Actual	Model
***Independently calibrated:***					
Discount factor	*β*	0.95	Average credit debt	0.05	0.05
Social stigma	Θ	-0.57	Credit card charge-off rate	5%	5%
Death rate of household heads	*ω*	0.06	Fraction of homeowner with mortgage	0.67	0.66
***Jointly calibrated:***					
Emotional attachment to the house	*δ*	0.152	Mortgage charge-off rate	0.15%	0.15%
Exponential mean Parameter	*μ*	1.38	Homeownership rate	0.66	0.66

In the mortgage foreclosure literature, the discount factor β is either calibrated or borrowed from the literature. Recent values range from 0.9 [23] to 0.94 [9] and 0.96 [7]. In previous work, households were classified as either “patient” or “impatient” with discount factors of 0.995 and 0.925, respectively [26]. If rational agents in the model are more impatient, then they will smooth their current period consumption by accumulating more unsecured debt during periods of unemployment. Here, we find that β = 0.95 works well to match the average credit card debt per household in the 2007 Survey of Consumer Finance. The value of the bankruptcy stigma factor θ was set to -0.57 so that the annual average charge-off rate of credit card debt is 5%, which is pretty standard in related literature [14]. The death rate of the household head is set to ω = 0.06 whereby the fraction of homeowners with a mortgage in the model matches the value (0.67) in the Survey of Consumer Finance.

In addition, agents with higher emotional attachment to a house *δ* will be more likely to purchase or keep a house, while worthy agents with higher exponential mean parameter *μ* are more reluctant to make a strategic decision to change states. Because mortgage foreclosure behavior is the primary goal of our study, these two parameters, which are closely related to housing strategic decisions, are calibrated jointly using an on-line multi-objective optimization. This on-line optimization keeps updating both parameters while the simulation is running until both the mortgage charge-off rate and homeownership rate meet their objectives. The average homeownership rate from 1989 to 2013 was 66%. The Federal Reserve Bank has published the charge-off rate on single family residential mortgages quarterly since 1991. The historical average of this rate from 1991 to 2006 is 0.145. Values of δ = 0.152 and μ = 1.38 serve to accurately calibrate the model with regard to the homeownership rate and the charge-off rate on mortgages.

#### Calibrated model fit

To estimate the model’s solution given stochastic shocks, representative agents are simulated until reaching a steady state and then for 200 periods afterwards. This is then repeated one million times in a Monte Carlo experiment. The presented results are the average of those one million resulting economic paths. The aggregate results for some variables that were not directly controlled for in the calibration are compared with empirical data in Table 3.

**Table 3:** Validation of the calibrated model.

	Model	Actual	Source
*In the normal economy*:			
Bankruptcy filing number per 100,000 household	682	614	Non-business bankruptcy, ABI (2006,2007)
Annually foreclosure rate (per 1k home)	3.74	2.45	National foreclosure rate, Zillow (2004~2006)
Homeownership rate of bankrupt household	50%	50%	(Zhu, 2011) and BAPCA chapter 7
Home equity of bankrupt household	0.14	0.21	(Miller, 2011)
Fraction of households with credit card debt (%)	48.33	38.1	Credit card balance, SCF 2013
*In the recession economy*			
Bankruptcy filing number per 100,000 household	783	1130	Non-business bankruptcy, ABI(2008~2013)
Credit card charge-off rate (%)	9.46	9.43	Credit card loans, All commercial banks, FRB 2009~2010

Note: ABI: American Bankruptcy Institute, BAPCA: Bankruptcy Abuse Prevention and Consumer Protection Act

FRB: Board of Governors of the Federal Reserve System, SCF: Survey of Consumer Finances

As can been seen from Table 3, the homeownership rate of bankrupt households in our model matches the data. The model also matches bankruptcy filings very well in the normal economy; however, the steady state bankruptcy filings in our recession economy are considerably lower than the real data during the 2007–2009 recession. This discrepancy likely indicates that the combination of high unemployment and declining house prices caused the bankruptcy rate during the recent recession to elevate much more than from either of those shocks individually. The model also slightly over-predicts the fraction of households with credit card debt. This result is not surprising because the only unsecured consumer loan that households can access in the model is a credit card loan. Additionally, the foreclosure rate in the model is 50% higher than national data from 2004 to 2006, but the house market price in that period was climbing instead of remaining constant as assumed in the steady state. The steady state model under-predicts the home equity of bankrupt households at 0.14 compared to 0.21 in [27], which included bankruptcy cases under any Chapters. In reality, households with low home equity tend to declare bankruptcy under Chapter 7, while those with high home equity can still file under Chapter 13 to keep their properties. In a theoretical sense, [1] proved that if a household has only the exempt asset (house), then it will never choose to file for Chapter 13 bankruptcy. Because our model only allows Chapter 7 bankruptcy, it is not surprising that the home equity is smaller than reality. Last but very importantly, the credit card charge-off rate in the recession economy perfectly matches the charge-off data in recession, which strongly supports the soundness of the setting and assumptions of the model economy.

Overall, the model performs well in accounting for non-targeted moments in the data. This model fit provides some model validation before proceeding to the policy analysis simulations.

## Model results

Having established that the model fits the data well in a steady state simulation with the calibrated parameters, we now turn to analyzing the two policies of interest: the down payment required to buy a house and the length of the credit exclusionary period. We do this with two separate sets of simulations. First we investigate the effect of changing the down payment and credit exclusionary period within a steady state environment, involving four different down payment levels and five lengths for the credit exclusionary period. Then we repeat that exercise in a dynamic environment where house prices follow a boom-and-bust pattern designed to match the recent American real estate market.

### Policy simulations with a steady state economy

Before focusing on the results of our simulation for the two key policy levers analyzed, a brief summary of the results to demonstrate the credibility of the model is worthwhile. (Results for many variables are in Tables A and B in S1 Appendix, but fuller results are available from the authors.) We find that unemployed renters are about four times more likely to declare bankruptcy than employed renters (3.47% versus 0.83%). This finding is comparable with other empirical studies of non-business bankruptcy [28]. It is also intuitive that the net saving levels of bankrupt renters are lower than the average levels for all renters. These two findings support the existence of two triggers for the bankruptcy of renters: heavy indebtedness and job loss. These same triggers were also found in a recent study of credit card default [14].

In general, the steady state model results make sense and match the real world. Renters tend to purchase a house when they are better off financially and have a job; we find a 5.78% versus 3.01% home buying rate, respectively, for employed versus unemployed renters. Compared to renters, homeowners have a lower bankruptcy rate, accounted for by the options of selling their house or going through foreclosure as alternative methods of alleviating financial distress. Years of homeownership (equivalent to relative equity), the level of savings, and house prices all affect the homeowner’s strategic decision. In any year, the bankruptcy probability is higher for an unemployed homeowner while the probability of mortgage foreclosure is also slightly higher among unemployed homeowners, which is consistent with our results in Table A in S1 Appendix and those in other empirical studies [29, 30]. Expected house prices have very limited impact on bankruptcy rates but a much higher effect on the rate of mortgage foreclosure. This indicates that home equity is a crucial determinant of a homeowner’s mortgage foreclosure. Homeowners will not choose foreclosure after 10 years of paying their mortgage but will still file for bankruptcy after 15 years. After 20 years, there are infinitesimal numbers of bankruptcy cases because home equity is almost always higher than the exemption, and the net gain from bankruptcy is very small. In reality, a household would prefer to borrow against its home equity.

Homeowner bankruptcies and mortgage default/foreclosure can be substitutes in some contexts and complements in others [31]. Bankruptcy and mortgage default can appear to be complements because they share two common causes: unemployment and hefty indebtedness. On the other hand, the substitution effect likely arises due to a household’s optimally choosing between these two strategic behaviors. “Homeowners in foreclosure who file for bankruptcy are 70% less likely to go through foreclosure and the time to foreclosure auction is significantly prolonged [32]. A rational homeowner will tend to choose foreclosure over bankruptcy when home equity is low, house price is low, or bankruptcy cost is high. This substitution effect can be used to explain several findings in the following discussion.

#### Down payment ratio

Table A in S1 Appendix summarizes the steady state statistics of model simulation using different down payment ratios (*D*) for a wide variety of variables. Besides the base case (10%), down payments equal to 20%, 5%, and 0% of the home price were simulated. As can been seen, when *D* = 20%, the mortgage charge-off rate declines 94% from the base case. Meanwhile, when *D* = 5% or 0%, the mortgage charge-off rate increases by factors of three and seven, respectively, from the base case. The annual foreclosure rate and total foreclosure numbers in the model follow the same trend, but change even more dramatically. These results suggest the easiest policy option to prevent another surge of mortgage foreclosures if home prices again is to increase the required down payment when buying a house.

This is consistent with the finding of a recent life-cycle model study [8]. The most straightforward side effect of a low down payment is stimulating home purchases [26]. As shown in Table A in S1 Appendix, the rate of home purchases increases when the down payment declines although less responsively than the foreclosure and mortgage charge off rates. Also, renters who purchase a house have a lower average saving level when the down payment required is low, suggesting the channel for the increased foreclosures to come.

Finally, the behavior of bankruptcy is also very interesting. Homeowner bankruptcy significantly decreases with a decrease in down payment ratio. However, this is mostly explained by the rise in foreclosures serving as a substitute. A lower down payment reduces the cost of foreclosure, so a financially distressed homeowner will more often choose mortgage foreclosure over bankruptcy. This behavior has also been supported by both theoretical work [5] and empirical results [33, 34].

#### The credit exclusionary period

Table B in S1 Appendix presents the full simulation results of the household responses to credit exclusionary periods of different lengths (3, 5, 7, 10, and 15 years). Most previous studies of credit default have selected the credit exclusionary period based only on empirical data or an assumption: for example, 4 years in mortgage foreclosure [35], 7 years in credit card default [14]. However, very few existing studies have investigated its effect on mortgage foreclosure and bankruptcy (one exception is [8]. As the penalty years in our model decrease from 15 years to 3 years, the mortgage charge-off rate increases by 28%. That is not unresponsive, but is much smaller than the magnitude of the impact found with changes in down payments. Correspondingly, the foreclosure rate and number increase by similar amounts. Unlike the down payment ratio, the bankruptcy rate changes little as the penalty years decrease. The intuition for this result is straightforward: both mortgage foreclosure and bankruptcy behavior will give rise to a strict liquidity constraint on the unworthy household in the following credit exclusionary period, which imposes a cost on these two strategic behaviors [36]. However, our simulations suggest this cost is low, so the effect is small.

Prolonging the credit exclusionary period produces much smaller benefits than increasing the down payment required to buy a house in terms of reducing bankruptcies and foreclosures while at the same time the longer credit exclusionary period comes with a profound social cost. Our results suggest that the reduction in adverse credit events from increasing the punishment that follows those events may not be worth the social and human cost. Thus, policy makers may wish to explore reducing the length of the credit exclusionary period.

### Policy simulations with a dynamic economy

**Fig 4:**
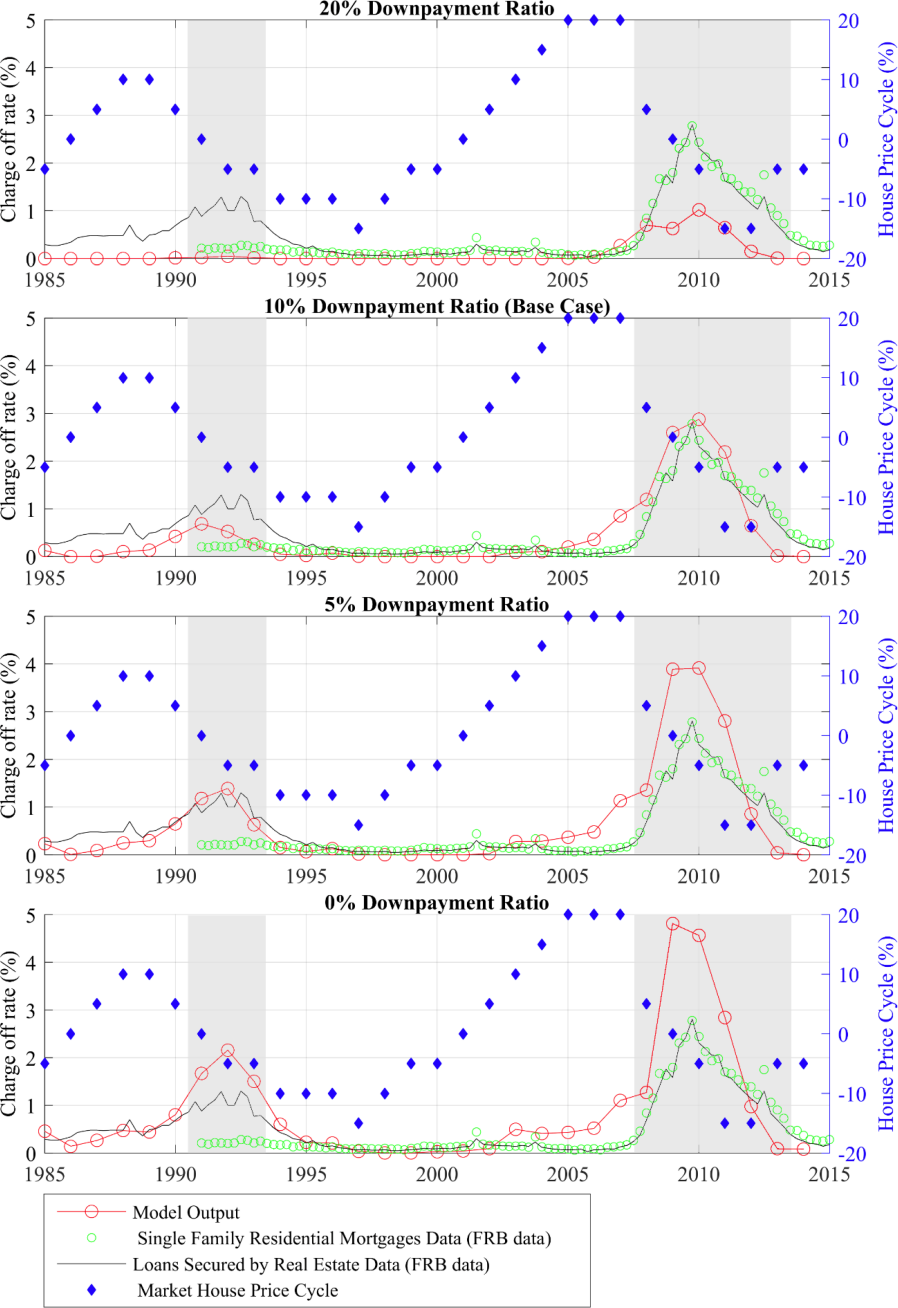
Mortgage charge-off rate with different downpayment ratios between 1985–2015: Model vs. data. Source: Author calculations.

We also examined the dynamics of the mortgage charge-off rate when the model economy and house price path replicate the United States’ recent economic condition (1985 to 2014). Previously work assumed three levels of house prices and simulated the history of the housing market by increasing the house price to a high level from 1999 to 2006 and setting it back to the medium level in 2007 in their model [2]. As a more precise and elaborate simulation, we set the house price dynamics in our model to the discrete cyclical component of the historical US Case-Shiller Home Price index. Then, all years with any months with an unemployment rate higher than 7% were defined to be in a recession state. In our simulation that means the years 1991–1993 and 2008–2013 are set to the recession state, with practical meaning in the model that there is more unemployment and the expected length of a spell of unemployment is longer. Using the above described Monte Carlo method, we simulate the stochastic model with these two exogenous variables and plot the simulated history of the aggregate mortgage charge-off rate in the second panel of Fig 4 (the base case). Fig 4 compares our model output with two series—the single family residential mortgage charge-off rate and the all real estate loan charge-off rate—for all U.S. commercial banks, as obtained from the Federal Reserve Bank of St. Louis, Federal Reserve Economic Data. As can been seen, house prices start to drop at the end of 2007, and the charge-off rate from both sources starts to rise in the same year.

The mortgage charge-off rate in our model starts to rise slightly one year earlier than the data. This could result from homeowners placing a higher option value on waiting to see if their situation improves than our model can match or might reflect that banks slowed foreclosures more than normal because of legal issues and the costs associated with it [37]. After its peak, the model value declines more quickly than the historical value (assumedly, because it went up faster). Meanwhile, both the timing and peak level of the charge-off rate from our model match those of the historical data in the late 2000s. Furthermore, the house price decline from 1989 to 1994 was also accompanied by an elevated charge-off rate on loans secured by real estate. The peak of the mortgage charge-off rate in the 1990s also can be observed in our model simulation output. As a comparison, in the early 1990s house price inflation only fell from +10% to -10%, whereas in the late 2000s it plummeted from +20% to -15%. It is very probable that the almost three times higher mortgage charge-off rate in the recent crisis was caused by the more drastic price drop. Similarly, [23] provides evidence from his dynamic simulation to show the importance of aggregate house prices during the recent mortgage market meltdown. Besides house prices, the longer recession period in the late 2000s also seems to have influenced homeowner foreclosure behavior.

In the simulation, we focus on the path of aggregate bankruptcies after 2005. Data before 2005 are not comparable to the later data because of the 2005 bankruptcy reforms which made bankruptcy filing more difficult and costly for homeowners [38, 39]. We find bankruptcies and foreclosures to move together. The complementary relationship between bankruptcy and default can be explained at the micro level and macro level. Individuals who file for bankruptcy and those who default on their mortgages tend to share two traits: unemployment and over-indebtedness. In aggregate, declines in house values increase the propensity of homeowners to default or file for bankruptcy in this dynamic simulation and in the data.

The overall performance of our dynamic experiment provides ample evidence that the model can accurately represent and predict aggregate U.S. mortgage foreclosure and bankruptcy behavior during periods of recession and declining house prices.

#### Down payment ratio

Whether the extremely low down payments of so many buyers in the early 2000s led to the mortgage meltdown has been debated in the popular press and academic literature [40, 41, 42]. Data for FHA and GSE purchased loans to show that the percentage of high leverage mortgages, defined as those with loan-to-value ratios higher than 95%, was about 1% in 1990 but rose to almost 40% in 2007 [43]. In the view of many scholars, this increasing share of low home equity mortgages was a major factor in the recent U.S. mortgage crisis.

Fig 4 presents the paths of the mortgage charge-off rate for the four down payment levels (10% was the baseline for the results discussed above). With a 20% down payment requirement, the major peak in 2010 is reduced from 3% in the baseline to 1%, and the minor peak in 1993 disappears. In contrast, in the 5% and 0% down payment ratio scenarios, the major peaks in 2010 jump to 4% and 5%, respectively. According to these results, a high down payment requirement can significantly dampen the burst of mortgage defaults in real estate market busts. This result is consistent with the findings from the steady state model and some previous findings on foreclosures during the crisis. For example, [2] find the larger fraction of high-leverage loans due to relaxed mortgage underwriting standards that emerged prior to the crisis explains 60% of the increase in the foreclosure rate. In [44], a stricter down payment limit significantly lowers the mortgage default rate, and combining recourse mortgages and loan-to-value limits makes the mortgage default rate less sensitive to fluctuations in aggregate house prices.

#### Credit exclusionary period

**Fig 5:**
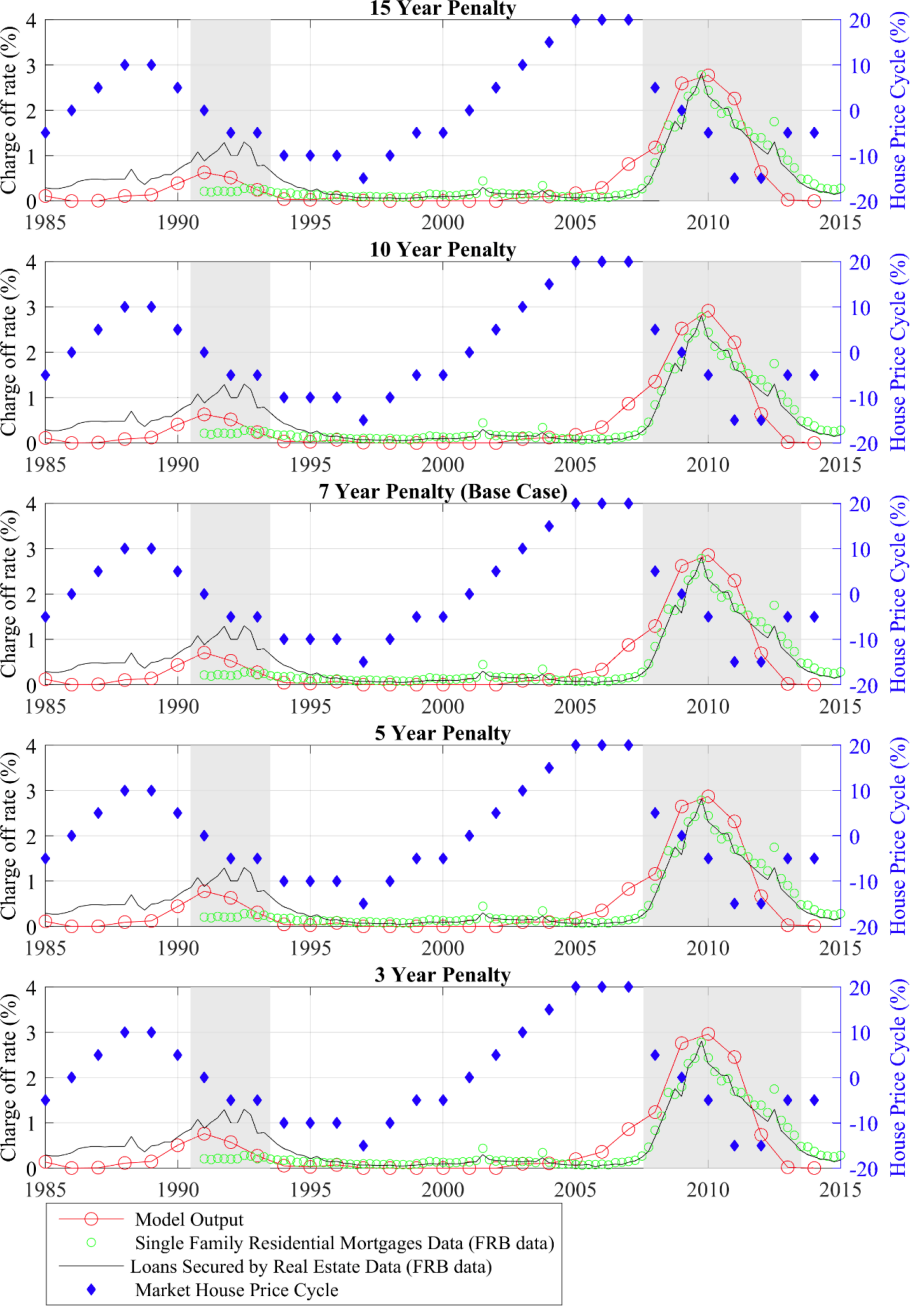
Mortgage charge-off rate with different lengths of credit exclusionary period between 1985–2014: Model vs. data. Source: Author calculations.

Fig 5 presents the results of the credit exclusionary period experiment. Visual inspection reveals almost no difference between the five paths from the 3-year penalty to the 15-year penalty scenarios. The credit exclusionary period has only a trivial effect on a household’s mortgage foreclosure behavior, especially in a period of falling house prices.

## Conclusions and policy suggestions

This paper employs a new, more general heterogeneous agent model of real estate and unsecured credit markets with stochastic shocks to employment and asset values (home prices) to investigate the ability of two policy levers to mitigate defaults in periods of recession and declining house prices. We demonstrate the ability of such a model to calibrate to empirical data and to simulate dynamic situations such as the recent recession and financial crisis. Our model can match both key long-run features and crisis characteristics of U.S. personal bankruptcy and residential housing mortgage foreclosure data. Given the observed path of house prices and other economic variables, the simulation of this model suitably matches the path of mortgage charge-offs from 1985 to 2014.

We provide evidence that the decline in housing prices combined with the large number of homeowners with little to no home equity was the major reason for the explosion of foreclosures and also contributed to elevated bankruptcy and credit card charge off rates. Our model confirms that for individuals there is a substitution effect between bankruptcy and mortgage foreclosure; however, from the aggregate point of view, an upward trend of both the bankruptcy filing rate and mortgage charge-off ratio existed simultaneously in the recent crisis. We also show that job loss is an important contributor to credit defaults (both credit card and mortgage). Further, we do all this in a model of optimizing agents, proving that in a stochastic environment you do not need non-optimal behavior or shortsightedness to explain the default patterns observed in the recent crisis; optimal behavior in a stochastic environment can reproduce the aggregate data.

A high down payment is shown to be very effective in reducing mortgage defaults in both steady state and boom-bust environments. Unfortunately, apparently having learned little from our recent experience, Fannie Mae and Freddie Mac reduced minimum required down payments in late 2014 from 10% to 3% for some qualified loans. If we fail to draw enough lessons from the past and keep encouraging homeowners to buy houses with very low home equity, it will be very hard to prevent future mortgage crises.

As another major contributions of this paper, we proved that the credit exclusionary period after a credit default is an ineffective punishment, inefficient at preventing foreclosures or bankruptcies. Given the small deterrent effect of being denied credit for longer periods of time, and in consideration of its high social cost, policy makers should seriously consider legislation to limit the allowable time period that defaulters are excluded from credit markets.

These two major findings make clear that it is far more effective to minimize mortgage defaults *ex ante* by requiring down payments of 10 or 20 percent (amounts that were traditionally normal until the past few decades) than to pressure people once they are in financial difficulty with sticks such as credit exclusionary periods. In the mortgage market, an ounce of prevention is, in fact, superior to a pound of cure.

## Supporting information

S1 Appendix Value functions and budget constraints. Table A: Impact of the Down Payment Ratio in a Steady State. Table B: Impact of the Credit Exclusionary Period in a Steady State.
(DOCX)
