# Supplementary material for: Reducing residential mortgage default: Should policy act before or after home purchases?
Source: PLoS One. 2018 Jul 19;13(7):e0200476. doi: 10.1371/journal.pone.0200476 (PMC6053153; doi:10.1371/journal.pone.0200476)
Supplement: S1 Appendix — Table A: Impact of the Down Payment Ratio in a Steady State. Table B: Impact of the Credit Exclusionary Period in a Steady State. (DOCX) [file pone.0200476.s001.docx]

## Supporting Information: Value Functions and Budget Constraints

Each household in this model maximizes a state-contingent value function of a current state variable over an infinite time horizon. The agent’s dynamic decision problem is characterized by a Bellman Equation which is subject to a budget constraint. The specifics of these value functions and budget constraints vary by the state of the agent. For our seven possible states, they are:

### Worthy Renter Value Function

Consider the problem of a renter who does not own a house. Her value function is denoted by $V^{R}$:

|  | $V^{R}\left( s;i; j=0 \right)=\max_{c} \left\{ u(c)+\beta\sum_{i^{'}} p_{{i,i}^{'}}V^{R}(s^{'};i^{'};j'=0) \right\}$ | (S1) |
| --- | --- | --- |

subject to

$$\frac{s^{'}}{1+r}+c+\xi\hat{H}=s+y\left( i \right)$$

$$s^{'}\geq-b$$

$$r=\left\{ \begin{matrix} r_{b} & s'<0 \\ r_{s} & s'\geq0 \end{matrix} \right..$$

In the above equations, $s$ is the end of period net asset balance, $i$ is the employment state, and $j$ is used to denote how many years a foreclosure or bankruptcy agent has been in an unworthy state (thus *j* = 0 here). Here $s^{'},i^{'},j^{'}$are all next period state variables, $c$ is the consumption in the current period, $\beta\in\left( 0, 1 \right)$is the household's per-period discount factor, and $r_{b}$ and $r_{s}$are the borrowing and saving interest rate, which need not be equal. In the value function,$u(x)$ is a twice continuously differentiable utility function of current consumption, with $u^{'}>0$, $u^{''}>0$, $u^{'}\left( 0 \right)=\infty$, and constant relative risk aversion ($\alpha$). The annual rental cost $\xi\hat{H}$ is proportional to the underlying value of housing ($\hat{H}$).

Most importantly, the annual income $y\left( i \right)$ is a function of the employment state in both a recession and the normal economy. In this setting, *ex ante* homogenous households are all facing *ex post* heterogeneous income shocks from their employment states. The employment state of each household independently and stochastically follows a two-state discrete time Markov chain. The transition probability matrix of this Markov chain depends on the underlying economy,

$$℘|economy=\left[ \begin{matrix} p_{00} & p_{01} \\ p_{10} & p_{11} \end{matrix} \right]$$

$p_{{i,i}^{'}}$ is the probability that the agent’s employment state will be $i^{'}$ in the next period, given the employment state is $i$ in the current period.

### Bankruptcy Unworthy Renter Value Function

After filing for bankruptcy, a renter will be excluded from the credit and mortgage markets for $\tau$ years with an unworthy flag ($j>0$). Thus, τ is the credit exclusionary period, one of the policies we specifically investigate. His value function is denoted by $V^{R,B}$.

|  | $V^{R,B}\left( s;i;j \right)=\max_{c} \left\{ u\left( c \right)+\beta\sum_{i^{'}} p_{{i,i}^{'}}V^{R,B}\left( s^{'},i^{'},j+1 \right) \right\}$  $\forall j\in\left\{ 1,2,3,4,5,\ldots,\tau-1 \right\}$ | (S2) |
| --- | --- | --- |

subject to

$$\frac{s^{'}}{1+r_{s}}+c+\xi\hat{H}=s+y\left( i \right)$$

$$s^{'}\geq0.$$

When $j=\tau$, the unworthy renter, foreseeing the return to a worthy state in the next period, uses the next period value function for a worthy renter from equation (1) above.

### Worthy Homeowner with No Mortgage Value Function

Now, consider the problem of homeowners who have paid off their mortgages. They will live in their own houses until the shock of forced sale (death). Denote the value function by $V_{k}^{h} (k>30)$:

$$V_{k}^{h}\left( s;i; j=0 \right)=\max_{c} \begin{aligned} \left\{ u\left( c \right)+\beta\left[ \omega\eta\sum_{H^{'}} q_{H,H^{'}}u\left( \left( 1-\chi\right)H^{'}+s^{'}+y(i) \right)+\left( 1+\omega\right)\sum_{i^{'}} p_{{i,i}^{'}} V_{k+1}^{h}\left( s^{'};i^{'};j'=0 \right) \right] \right\} \\ \forall k>30 , \forall j\in\left\{ 1,2,3,4,5,\ldots,\Gamma-1 \right\} \end{aligned}$$

|  |  | (S3) |
| --- | --- | --- |

subject to

$$\frac{s^{'}}{1+r}+c+\kappa\hat{H}=s+y\left( i \right)$$

$$s^{'}\geq-\left( 1+r \right)b.$$

Here, $H$ and $H'$ are house market prices in the current and next period. As detailed below, the agent’s house price state is modeled here as a nine-state discrete time Markov chain whose transition probability matrix $\mathbb{Q}$ is empirically calibrated to historical house prices. The prices in each state are a certain percentage higher or lower than the underlying value of housing ($\hat{H}$). Thus $q_{H,H^{'}}$ represents the probability that next period’s house price is $H'$ given the current market price is $H$. One benefit of homeownership is the avoidance of rental costs, but ownership still incurs an annual maintenance cost$\kappa\hat{H}$. In each period, homeowners with no mortgage are forced to sell their houses due to the death and change of the household head with probability $\omega$. The importance of the bequest motive is modeled by the parameter $\eta$. The non-foreclosure sale of the house incurs a proportional cost ${\chi H}^{'}$ (for example, a real estate agent’s commission and moving expenses).

### Bankruptcy Unworthy Homeowner with No Mortgage Value Function

Similar to the unworthy renter, after filing for bankruptcy, homeowners with no mortgage will also be excluded from the credit market for $\tau$ years with an unworthy flag, $j>0$. The value function is denoted by$V^{h,B}$.

$$V_{k}^{h,B}\left( s;i;j \right)=\max_{c} \begin{aligned} \left\{ u\left( c \right)+\beta\left[ \omega\theta\sum_{H^{'}} q_{H,H^{'}}u\left( \left( 1-\phi\right)H^{'}+s^{'}+y(i) \right)+\left( 1+\omega\right)\sum_{i^{'}} p_{{i,i}^{'}} V_{k+1}^{h,B}\left( s^{'};i^{'}; j+1 \right) \right] \right\} \\ \forall k>30, \forall j\in\left\{ 1,2,3,4,5,\ldots,\tau-1 \right\} \end{aligned}$$

|  |  | (S4) |
| --- | --- | --- |

subject to $\frac{s^{'}}{1+r}+c+\kappa\hat{H}=s+y\left( i \right)$

$$s^{'}\geq0.$$

Again, in the final period of the credit exclusionary period, the one-period-ahead value function in (4) is replaced with the one for a worthy homeowner with no mortgage.

### Worthy Homeowner Value Function

Now, let us consider the decision problem of homeowners who live in their own homes, but have not paid off their mortgages. Their value functions are denoted by$V_{k}^{h} (0<k\leq30)$

|  | $V_{k}^{h}\left( s;i; j=0 \right)=\max_{c} \begin{aligned} \left\{ u\left( c \right)+\beta\sum_{i^{'}} p_{{i,i}^{'}} V_{k+1}^{h}\left( s^{'};i^{'}; j'=0 \right) \right\} \\ \forall0<k\leq30 \end{aligned}$ | (S5) |
| --- | --- | --- |

subject to

$$\frac{s^{'}}{1+r}+c+\kappa\hat{H}+\Psi(h,D,r_{m}) =s+y\left( i \right)$$

$$s^{'}\geq-\left( 1+r \right)b.$$

In addition to paying the annual maintenance cost$\kappa\hat{H}$ these homeowners have the obligation to pay their mortgage in a total annual amount of $\Psi(h,D,r_{m})$. Upon granting a mortgage loan for the home purchase, the lender requires all borrowers to make a down payment, which is expressed as a percentage ($D$) of the house value. The annual mortgage payment is dependent on *D*, the house price when purchased ($h$) and the mortgage interest rate ($r_{m}$). Note that while the mortgage payments are represented by an annual sum for simplicity, the calibrated model when solved computes the payment amounts based on monthly payments and daily compounding of interest.

### Bankruptcy Unworthy Homeowner Value Function

If the homeowner files for bankruptcy, he will be excluded from borrowing unsecured debt for a period of τ years. If the bankruptcy trustee does not sell his house, the unworthy homeowner will keep paying the mortgage until the end of the repayment plan. The value function is denoted by $V_{k}^{h,B} (0<k\leq30)$

|  | $V_{k}^{h,B}\left( s;i;j \right)=\max_{c} \begin{aligned} \left\{ u\left( c \right)+\beta\sum_{i^{'}} p_{{i,i}^{'}} V_{k+1}^{h, B}\left( s^{'};i^{'}; j+1 \right) \right\} \\ \forall0<k\leq30, \forall j\in\left\{ 1,2,3,4,5,\ldots,\tau-1 \right\} \end{aligned}$ | (S6) |
| --- | --- | --- |

subject to

$$\frac{s^{'}}{1+r}+c+\kappa\hat{H}+\Psi(h,D,r_{m}) =s+y\left( i \right)$$

$$s^{'}\geq0.$$

As before, the future value function for a worthy homeowner is substituted into (6) in the final period of credit unworthiness.

### Foreclosure Unworthy Renter Value Function

A renter who has been through a foreclosure within the last $\tau$ years is not allowed to buy a house or obtain a new mortgage. Meanwhile, this renter cannot accumulate more unsecured debt, which means he has to use his current income to pay his interest expense and consumption in each period. The value function is denoted by$V^{R,F}$.

|  | $V^{R,F}\left( s;i;j \right)=\max_{c} \left\{ u(c)+\beta\sum_{i^{'}} p_{{i,i}^{'}}V^{R,F}(s^{'};i^{'};j+1) \right\} \forall j\in\left\{ 1,2,3,4,5,\ldots,\tau-1 \right\}.$ | (S7) |
| --- | --- | --- |

When $j=\tau$, the agent is automatically returned to the state of a worthy renter as specified above in the next period and solves its value function accordingly.

S1 Table A. Impact of the Down Payment Ratio in a Steady State

|  |  | Downpayment Ratio (%) | | | |
| --- | --- | --- | --- | --- | --- |
|  |  | 20 | 10 (base) | 5 | 0 |
| Mortgage Charge-off Rate (%) | | 0.006 | 0.149 | 0.462 | 1.034 |
| Annually Foreclosure Rate (1k Home) | | 0.19 | 3.74 | 13.44 | 68.61 |
| Charge-off Rate on Credit Card (%) | | 7.5 | 5.05 | 4.1 | 4.18 |
| Annual Homeowner Foreclosure | | 6.18 | 248.37 | 829.07 | 2453.2 |
| Annual Homeowner Bankruptcy | | 229.09 | 341.2 | 156.86 | 37.53 |
| Annual Renter Bankruptcy | | 390.13 | 341.32 | 321.26 | 250.25 |
| Renter Annual home purchase ratio (%) | Employed | 1.14% | 5.95% | 7.39% | 7.15% |
|  | Unemployed | 0.54% | 1.26% | 2.21% | 4.29% |
| Renter Annual Bankruptcy Ratio (%) | Employed | 0.47% | 0.98% | 0.90% | 0.45% |
|  | Unemployed | 3.15% | 4.57% | 4.07% | 2.44% |
| Homeowner Annual Bankruptcy Ratio (%) | Employed | 1.11% | 0.70% | 0.32% | 0.11% |
|  | Unemployed | 2.15% | 3.01% | 1.67% | 0.74% |
| Homeowner Annual Foreclosure Ratio (%) | Employed | 0.03% | 0.57% | 1.88% | 8.90% |
|  | Unemployed | 0.05% | 1.13% | 4.87% | 14.48% |
| Annual Homeowner short sale Ratio (%) | Employed | 0.00% | 0.00% | 0.02% | 0.02% |
|  | Unemployed | 0.00% | 0.01% | 0.15% | 0.10% |
| Renter Net Asset Given Home Purchase | Employed | 0.686 | 0.265 | 0.212 | 0.166 |
|  | Unemployed | 1.271 | 0.955 | 0.549 | 0.208 |
| Renter Net Asset Given Bankruptcy | Employed | -0.236 | -0.246 | -0.249 | -0.245 |
|  | Unemployed | -0.179 | -0.196 | -0.197 | -0.190 |
| Homeowner Net Asset Given Bankruptcy | Employed | -0.314 | -0.200 | -0.199 | -0.202 |
|  | Unemployed | -0.282 | -0.164 | -0.164 | -0.142 |
| Homeowner Net Asset Given Foreclosure | Employed | -0.321 | 0.017 | 0.038 | 0.139 |
|  | Unemployed | -0.251 | -0.026 | 0.014 | 0.130 |

Note: 100,000 Households

S1 Table B. Impact of the Credit Exclusionary Period in a Steady State

|  |  | Credit Exclusionary Period (yrs) | | | | |
| --- | --- | --- | --- | --- | --- | --- |
|  |  | 15 | 10 | 7 (Base) | 5 | 3 |
| Mortgage Charge-off Rate (%) | | 0.131 | 0.140 | 0.149 | 0.157 | 0.168 |
| Annually Foreclosure Rate (Per 1k Home) | | 3.46 | 3.58 | 3.75 | 3.94 | 4.24 |
| Charge-off Rate on Credit Card (%) | | 5.12 | 5.07 | 5.05 | 5.09 | 5.13 |
| Annual Homeowner Foreclosure | | 219.96 | 233.77 | 248.59 | 263.44 | 284.33 |
| Annual Homeowner Bankruptcy | | 322.60 | 330.71 | 339.69 | 350.12 | 367.64 |
| Annual Renter Bankruptcy | | 327.88 | 338.03 | 342.78 | 348.48 | 347.12 |
| Renter Annual home purchase ratio (%) | Employed | 5.92% | 5.96% | 5.95% | 5.93% | 5.85% |
|  | Unemployed | 1.34% | 1.36% | 1.34% | 1.27% | 1.05% |
| Renter Annual Bankruptcy Ratio (%) | Employed | 0.98% | 0.99% | 0.99% | 0.98% | 0.95% |
|  | Unemployed | 4.59% | 4.60% | 4.51% | 4.56% | 4.39% |
| Homeowner Annual Bankruptcy Ratio (%) | Employed | 0.74% | 0.71% | 0.70% | 0.70% | 0.72% |
|  | Unemployed | 3.19% | 3.01% | 3.02% | 3.09% | 3.16% |
| Homeowner Annual Foreclosure Ratio (%) | Employed | 0.56% | 0.55% | 0.57% | 0.59% | 0.62% |
|  | Unemployed | 1.17% | 1.16% | 1.18% | 1.18% | 1.26% |
| Annual Homeowner short sale Ratio (%) | Employed | 0.00% | 0.00% | 0.00% | 0.00% | 0.00% |
|  | Unemployed | 0.01% | 0.01% | 0.02% | 0.01% | 0.01% |
| Renter Net Asset Given Home Purchase | Employed | 0.390 | 0.327 | 0.266 | 0.213 | 0.141 |
|  | Unemployed | 1.445 | 1.193 | 0.958 | 0.733 | 0.470 |
| Renter Net Asset Given Bankruptcy | Employed | -0.246 | -0.246 | -0.246 | -0.247 | -0.246 |
|  | Unemployed | -0.195 | -0.196 | -0.196 | -0.196 | -0.196 |
| Homeowner Net Asset Given Bankruptcy | Employed | -0.199 | -0.199 | -0.199 | -0.199 | -0.200 |
|  | Unemployed | -0.164 | -0.164 | -0.163 | -0.164 | -0.164 |
| Homeowner Net Asset Given Foreclosure | Employed | 0.002 | 0.020 | 0.021 | 0.009 | -0.017 |
|  | Unemployed | -0.023 | -0.033 | -0.025 | -0.033 | -0.050 |

Note: 100,000 Households
